# Persistent and substantial impacts of the Deepwater Horizon oil spill on deep-sea megafauna

**DOI:** 10.1098/rsos.191164

**Published:** 2019-08-28

**Authors:** Craig R. McClain, Clifton Nunnally, Mark C. Benfield

**Affiliations:** 1 Louisiana Universities Marine Consortium, 8124 Highway 56, Chauvin, LA 70344, USA; 2 Department of Biology, University of Louisiana, Lafayette, 410 East St. Mary Boulevard, Billeaud Hall, Lafayette, LA 70503, USA; 3 Department of Oceanography and Coastal Sciences, College of the Coast and Environment, Louisiana State University, Baton Rouge, LA 70803, USA

**Keywords:** oil spill, environmental impacts, pollution, ecosystem health

## Abstract

The Deepwater Horizon spill is one of the largest environmental disasters with extensive impacts on the economic and ecological health of the Gulf of Mexico. Surface oil and coastal impacts received considerable attention, but the far larger oil spill in the deep ocean and its effects received considerably less examination. Based on 2017 ROV surveys within 500 m of the wellhead, we provide evidence of continued impacts on diversity, abundance and health of deep-sea megafauna. At locations proximal to the wellhead, megafaunal communities are more homogeneous than in unimpacted areas, lacking many taxonomic groups, and driven by high densities of arthropods. Degraded hydrocarbons at the site may be attracting arthropods. The scope of impacts may extend beyond the impacted sites with the potential for impacts to pelagic food webs and commercially important species. Overall, deep-sea ecosystem health, 7 years post spill, is recovering slowly and lingering effects may be extreme.

## 1. Introduction

On 20 April 2010, and continuing for 87 days, approximately 4 million barrels [1] spilled from the Macondo Wellhead (MW) as part of the Deepwater Horizon (DWH) accident making it the largest accidental marine oil spill in history. The biological impacts of the oil spill were severe [2–5], including in the deep sea, a habitat typically characterized by high biodiversity [6]. Although previous studies indicated some benthic recovery in deep-sea systems by 2014, diversity was nowhere near baseline levels [7–11]. Further information about how deep-sea biodiversity is recovering is urgently needed given the linkage between deep-sea biodiversity and multiple ecosystem functions and services [12], such as carbon sequestration, nutrient regeneration, microbial-based detoxification and deep-sea fish stocks [13]. Moreover, there is a great need to understand the resilience and recovery of deep-sea ecosystems in the face of predicted increases in deep-water exploration and extraction worldwide but particularly in the Gulf of Mexico (Gulf) [14–16].

Much of the DWH oil spill remained at depth. Oil released at 1511 m, plume dynamics, and the 2.9 million litres of dispersant injected directly into the source [17,18] led to 35% of the hydrocarbons being trapped and transported in deep-sea plumes [19]. These deep, subsurface plumes consisted of a mixture of hydrocarbons [20,21]. Weathering, burning and application of dispersant to sea surface oil also returned additional hydrocarbons to the deep-sea floor as oiled marine snow [21–23]. Toxic hydrocarbons, dispersants and heavy metals released in association with drilling and well control created chronically and acutely toxic conditions in its vicinity [6]. Parts of the deep Gulf sea floor became a ‘toxic waste dump’ [6].

Impacts on deep-sea benthos were severe. Declines in benthic foraminifera density (↓80–93%) [7], copepod abundance (↓64%) [9], meiofaunal diversity (↓38%) [8], macrofaunal diversity (↓54%) [8] and megafaunal richness (↓40%) [11] occurred near the wellhead site within the first few months. One year later, the impacts on macrobenthic community diversity were still evident [10]. These negative impacts correlated with increases in total petroleum hydrocarbons (TPH), polycyclic aromatic hydrocarbons (PAH) and barium in deep-sea sediments [7–10]. In 2011, macrofaunal and meiofaunal richness were 22.8% and 28.5% less in the impact zone [24] than at unimpacted control sites, respectively, and correlated with PAH and TPH concentrations 40 and 34 times higher. Although in 2014, PAH and TPH concentrations decreased, PAH was still 15.5 and TPH 11.4 times higher in the impact zone versus the non-impact zone, and the impact zones still exhibited depressed meiofaunal and macrofaunal diversity [25]. Whereas corals have, and continue to receive, considerable attention (e.g. [26–29]), studies examining the impacts of the DWH oil spill on deep-sea biological communities ended in 2014. An assessment of coral health found an ongoing effect and the majority of colonies still had not recovered by 2017 [27]. These coral results suggest a lasting impact on sediment benthic communities. This post-2014 knowledge gap for impacts on the deep-sea sediment communities raises questions about the lasting effects on the ecosystem and organismal health in the deep Gulf.

Here, we examined the impact 7 years after the DWH spill on benthic megafauna. We examined ecosystem health by comparing alpha diversity, beta diversity and abundances immediately after the oil spill in 2010 and contemporaneously at both impacted and background sites in 2017.

## 2. Material and methods

### 2.1. ROV transect locations

Valentine & Benfield [11] conducted ROV video transects at 1498–1601 m in August and September 2010, one–two months post spill. Their sampling included sites 2000 m south (2000-S) and 500 m due north (500-N) of the well. They employed a radial survey design [11] that included 12 transects 250 m, beginning at 0° and separated by 30° increments at 2000-S. At the 500-N site, a series of nine 250 m long transects were surveyed from 90° to 270° at 22.5° increments. All transects at 500-N were conducted at bearings north of a line running east to west to avoid operating in a region where there was substantial debris from the DWH.

**Figure 1.**
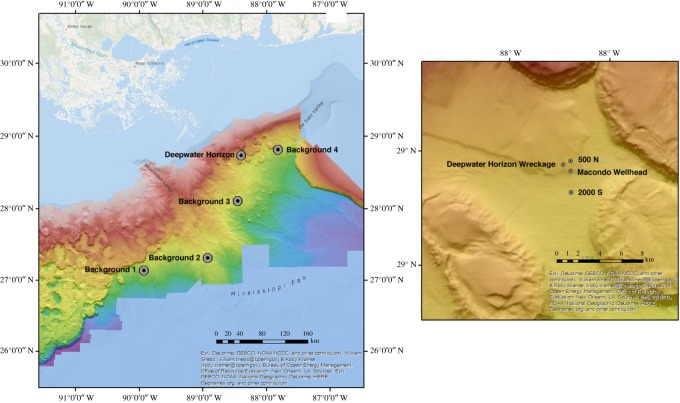
Map of background and impacted sample sites where video transects were conducted. The location of the DWH wreckage and Macondo wellhead are also shown.

On 1–2 June 2017, we replicated the 2010 radial transects at the 2000-S and 500-N sites. We compared these to transects taken in the Gulf at similar depths (1960–2179 m) at four additional control sites (figure 1; electronic supplementary material, table S1). Data for the video transects were available due to ongoing but non-related research of McClain at these sites. Each of the control sites was chosen to be approximately 100 km distant from each other.

### 2.2. ROV transect assemblage analysis

2017 video transects were performed with high-definition (HD) 4 K video cameras with Oceaneering's *Global Explorer* from the R/V *Pelican*. Megafauna were identified from all video transects to the species level where possible. Every effort was made to assign morphologically distinct organisms (i.e. morphospecies) with an appropriate Latin binomial.

Alpha megafauna diversity was calculated for video transect using the Shannon–Wiener index as defined as *H = −*sum *p_i_* ln(*p_i_*), where *p_i_* is the proportional abundance of species *i*. *H* was calculated using the vegan package [30] in R. Rarefaction curves were calculated for combined transects for each site × time combination using the iNEXT package [31,32] with interpolation and extrapolation of Hill number with diversity order equal to one. Abundance was the total number of individuals per transect. Linear mixed-effects models were constructed with transect group—DWH 2010, background 2017 and DWH 2017—as the independent variable with *H* or abundance as the dependent. Linear mixed-effects models were constructed with the lmer function in the package lme4 [33]. ANOVAs were analysed with the ANOVA function in the package car [34]. Sites within groups were treated as random effects in the models. *Post hoc* tests were conducted using Tukey honest significant differences test with the ghlt function in the package multicomp [35].

Total beta diversity was calculated on the Hellinger-transformed abundance as the total variance in a community data matrix [36] using the adespatial package [37] in R. The Hellinger transformation was implemented to preserve Euclidean distances between transects as advocated for when in use with redundancy or principal components analysis (PCA) [38]. Hellinger distance also offered a better compromise between linearity and resolution than some other distance metrics [38]. Total beta diversity was calculated for groups of transects including all 2010 DWH, DWH 2010 500-N, DWH 2010 2000-S, all 2017 DWH, DWH 2017 500-N, DWH 2017 2000-S, background sites 1, 2, 3 and 4. Total beta diversity was calculated as the total variance in a community data matrix.

A principal component analysis was conducted on the Hellinger pre-transformed data, using the function decostand and rda in the vegan package [30]. Compositional differences between groups were tested using the PERMDSP2 procedure for the analysis of multivariate homogeneity of group dispersions (variances) using the betadispr function in the vegan package [30] with a subsequent permutation test. In this method, the average multivariate distance of individual transects is calculated from the group centroid in the PCA space. To test if the dispersions of the three groups—DWH 2010, background 2017 and DWH 2017—were different, these distances were analysed with ANOVA.

## 3. Results

**Figure 2.**
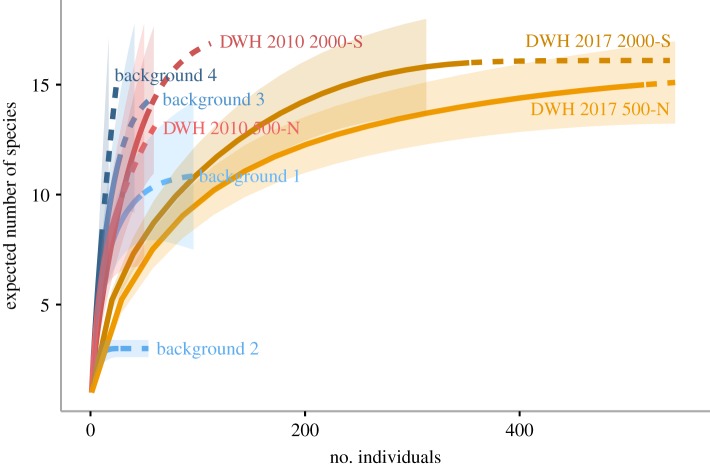
Rarefaction plot, expected number of megafaunal species versus the number of sampled individuals combined across transects, for each site × time. Solid lines represent interpolated rarefaction curves and dashed lines represent extrapolations of the curve.

**Figure 3.**
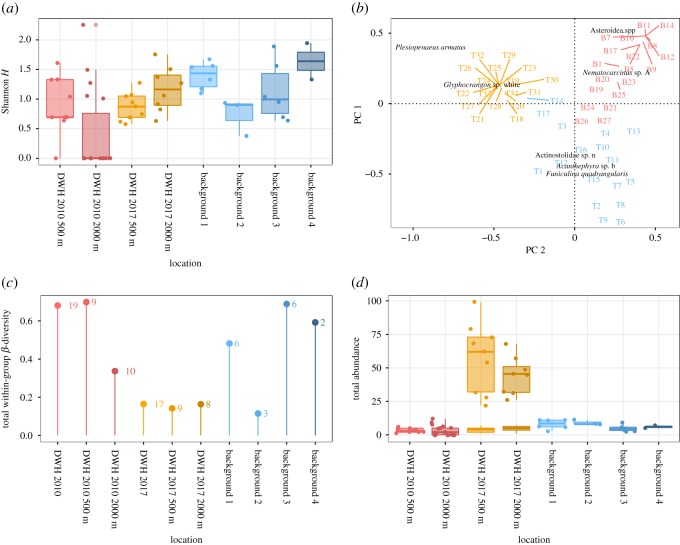
Analysis of megafauna communities on ROV video transects in the deep Gulf of Mexico. Transects from the DWH site from a 500 and 2000 m distance from the wellhead (in 2010 and 2017) are compared to four background sites (in 2017). (*a*) Boxplots of diversity as measured by Shannon–Wiener *H*′. Values for each transect are shown as jittered points. (*b*) PCoA of Hellinger-transformed data. Species distinguishing each cluster of points are provided. Numbers beginning with T indicate the transect number. Species loadings are only shown for those in the upper and lower 25% quantile of values. (*c*) Within-site beta diversity measured on Hellinger-transformed data. Numbers refer to the total number of transects in that group. (*d*) Boxplot of total abundances on the transect. Lower boxplots just for DWH 2017 are total abundances with arthropods removed.

Considerable differences exist in the diversity and abundance of megafauna among the 2010 DWH, 2017 DWH and 2017 background transects (figures 2 and 3; electronic supplementary material, tables S2 and S3). Diversity, as measured by the expected number of species, was similar between background sites and DWH sites in 2010 (figure 2). Although abundance was greater in DWH sites in 2017, the diversity was generally lower in both the 2017 background sites and 2010 DWH sites (figures 2 and 3). The lower diversity of background site 2 may reflect its highly disturbed location at the end of the Mississippi Canyon. Alpha diversity was at its lowest in 2010 at the oil spill site (Tukey HSD pairwise: *p* ≤ 0.001, 0.019*7*), driven by a considerable number of transects with no or little megafauna, particularly at the 2000-S site (figure 3*a*). Note the high estimates of diversity as calculated by rarefaction for the 2010 DWH do not account for these transects with less than or equal to 1 individual.

While diversity values for the 2017 DWH site were not significantly different from background sites (figure 3*a,* Tukey HSD pairwise: *p* = 0.5285), abundance and taxonomic composition of megafauna were substantially different (figure 3*b*–*d*). In terms of taxonomic composition, 2010 DWH, 2017 DWH and 2017 background transects exhibited significantly different faunas (figure 3*b*; electronic supplementary material, tables S4 and S5, permutation test for homogeneity of multivariate dispersions: *p* = 0.001). 2017 DWH transects exhibited little fauna turnover among them, i.e. the site was homogeneous in taxonomic composition. Beta diversity was lower than in both the 2010 DWH and 2017 background sites (figure 3*c*). This low beta diversity persisted even when the 500-N and 2000-S (2.5 km distance from each other) were grouped at the 2017 oil spill site. Indeed, the total beta diversity of 17 video transects at 2017 DWH site was near equal to that seen on just three transects at background site 2 (figure 3*c*).

Much of the beta-diversity differences among the sites were driven by large numbers of arthropods at DWH site in 2017 including the red shrimp *Nematocarcinus*, a white caridean *Glyphocrangon* shrimp and the commercially important crab *Chaceon quinquedens* (figure 3*b,d*). In 2010, compositional differences were driven by the increased numbers of starfish and *Nematocarcinus* sp*.* and at background sites by Actinostolidae anemones, another red shrimp *Acanthephyra*, and the sea pen *Funiculina quadrangularis*.

Abundances of megafauna at 2017 DWH were 7.5 times higher, ranging from 22 to 99 individuals (mean 51.4) compared to background ranges from 2 to 11 individuals (mean 6.8, ANOVA: *p* < 0.0001; electronic supplementary material, table S6 and S7). Increased arthropod abundance alone drove the differences in total abundances at the 2017 DWH compared to 2010 or background sites (figure 3*d*). Removing these arthropod abundances reduced the significance in the difference between sites (electronic supplementary material, table S8 and S9, ANOVA: *p* = 0.0971). Across all 2017 DWH transects, non-arthropod taxa made up just 69 individuals compared to the 801 arthropods. Moreover, three major taxonomic groups: asteroidea, sipuncula and platyhelminthes were entirely absent on the 2017 DWH transects despite being present at control sites.

## 4. Discussion

The area around the DWH wreckage site and MW continue to exhibit considerable deep-sea biological degradation after 7 years. These impacts include lower diversity, ecological homogeneity and abnormal population densities. Recovery times at the impacted site mimic the slow recovery rates of deep-sea faunas associated with protracted rates of physiological processes in deep-sea organisms, low fecundity and larval output, long generation times and the slow recovery of relatively stable, muddy sediments [39]. One background site did have diversity values close to the 2017 DWH site. Site 2 sits at the lower end of the Mississippi Canyon axis and probably experiences disturbance, which may explain the overall decreases in alpha- and beta diversity (figure 3). This suggests the diversity of the DWH site may be reflective of habitat affected by ongoing disturbance.

One potential explanation for the differences observed here in 2017 between the background and the DWH sites is depth differences between the sites. The transects at DWH range in depth from 1499 to 1591 m and the background transects are 1984–2178 m for a minimum of 393 m and a maximum of 679 m between the two groups. This sample design was an unfortunate outcome that reflects the research was unfunded and the dives at DWH were opportunistic. The background ROV transects were collected as part of another supported research project. However, the depth difference is unlikely to explain the results. First, the deeper background sites should exhibit lower, not the observed higher, alpha-diversity compared to the shallower DWH horizon sites [40]. Indeed, the high particulate organic carbon flux of this Gulf of Mexico region [40,41] should also drive diversity to be higher than the other deeper background sites [42–44]. Second, the abundances would be expected to be higher at the shallower sites [45,46]. And although abundances are higher at the shallower DWH site, increases in abundance would be expected to occur across megafaunal taxa. The result here shows increases are limited to arthropods. The differences in abundances between background and impacted sites are non-existent when arthropods are removed. This finding suggests that abundance was impacted by a process other than a simple bathymetric and energy availability relationship. Third, the substantial differences in taxonomic composition between the background and impacted sites suggest as well a mechanism beyond bathymetric differences. The 2017 background and DWH communities are less than 20% similar in composition to each other. The approximately 500 m depth difference between DWH and background sites is unlikely to account for a greater than 80% compositional change. This turnover percentage is more typical of a greater than 1000 m depth difference at much shallower depths [47]. Upper to lower middle slope communities often show greater than 40% similarity [48]. Indeed, research from the Gulf of Mexico indicates the compositional differences in communities at this depth and over this depth range would be minimal. Powell *et al.* [49] demonstrate that the deep-sea demersal fish of the northern Gulf of Mexico form a single compositional group from depths 1533–3075 m based on Bray–Curtis similarities and cluster analysis. Pequegnat *et al*.'s [50] classic paper shows that the megafauna of the Gulf of Mexico fall naturally into several depth zones including the ‘Upper Abyssal Zone’ from 1000 to 2275 m. These combined lines of evidence indicated the bathymetric differences between the DWH and background transects are insufficient to account for the differences observed here.

High species turnover, i.e. high beta diversity, across scales is a common feature of the deep-sea benthos [39,51]. The faunal homogeneity across transects of the 2017 DWH site stands in stark contrast to this pattern. Lowered beta diversity, compositional shifts and increased homogeneity are frequent in heavily polluted sites [52,53] with communities often converging on a limited number of opportunistic and pollution-tolerant species [9,52,54]. The lack of natural history information for deep-sea animals prevents the determination of species with ecological traits that enable them to be opportunistic or pollution tolerant; however, some patterns are notable. At 2017 DWH, large mobile megafauna common at background sites, particularly giant isopods, holothurians and sessile faunal, such as sea pens, fly-trap anemones and Venus flower basket sponges, were absent. The absence of these taxa may reflect low resiliency especially in cnidarians [55].

This increase in arthropod abundances at the 2017 DWH site may reflect a reef effect of the rig structure. Rigs are often reported as attractors of animals, especially fishes [56,57], greatly increasing abundances, although diversity and composition may be fundamentally different from natural hard substrates [56,57]. The rigs may provide rare hard habitat in an open ocean that may allow for range expansion [58,59]. Although the ‘rig and reef’ effect is poorly examined for deep-sea species, studies on deep-sea coral species have found them inhabiting rig structures [60,61]. Nevertheless, several observations argue against this ‘reef effect.’ The wreckage of the DWH occurs at the 500-N site with a concordant increase in arthropods. However, the 2000-S site also exhibits high arthropod abundances (figure 3*d*) but lacks any rig structure or debris. In addition, *Chaceon quinquedens*, in high abundance, is known to prefer silt over debris or structure from shipwrecks [62]. We also note observationally that there was a striking absence of fauna on the DWH wreckage. A high diversity of suspension feeders is typical on both natural [21,32] and anthropogenic hard substrates [63] in the deep sea. The fauna found at the 2017 DWH site in 2017 was limited to soft-sediment and demersal species.

We posit that degraded hydrocarbons at the DWH site are serving as both a chemical attractor while proving a toxin for other species. As recently as 2014, years before this study, sediment PAH concentrations were 15.5 times (218 versus 14 ppb) and TPH 11.4 times (1166 versus 102 ppm) higher in the impact zone versus the non-impact zone [25]. Lack of adequate sedimentation to cap oiled sediments was evident in ROV observations of a crab individual re-exposing oiled sediments as it walked. In addition, previous research found Macondo oil present in *C. quinquedens* both in 2010–2011 and again in 2014 [64]. The DWH arthropod abundances greater than 7.5 times that of the background also merit explanation. Attraction by crustaceans to hydrocarbons is a common behavioural response as specific oil components may mimic natural chemical cues [65]. *Homarus americanus* displayed feeding attraction to kerosene and its branched-cyclic and polar-aromatic fractions at low concentrations [66] and readily consumed fish heavily contaminated with bunker oil [67]. The affinity of *H. americanus* to hydrocarbon attraction was proposed as the cause of the mass mortality of the species in the Buzzard Bay oil spill [68]. In several crustacean species, cuticular hydrocarbons serve as sex hormones [69–71] suggesting the possibility of oil spill hydrocarbons as an attractant mimic. This provides a plausible mechanism that the increased abundance of arthropods at the 2017 DWH site may be due to an influx of individuals drawn by degraded hydrocarbons serving as a sex hormone mimic and attractant. The lack of increased arthropod abundances one to two months after the spill at the DWH may reflect insufficient time for hydrocarbon degradation to occur and provide the appropriate chemical cues or the fact that much of the fauna (indicated by several 2010 DWH transects with no or little diversity) had gone locally extinct.

Overall, our findings support the need for three main courses of action. First, we greatly urge for longer funding periods for assessing deep-sea environmental impacts beyond the typical 3–5-year funding cycles. Girard *et al*. [55] find that corals impacted by the DWH spill may take up to three decades for recovery. This study was only possible because opportunity and ship time were available on a non-related project. No agency is currently funding impact studies to the deep-sea benthos at the DWH spill site, with the last published impact assessments in 2014. The slow times in which deep-sea processes play out, including recovery times, indicate a definite mismatch between monitoring recovery and funding length.

Second, we urge for increased funding and commitment for pre-impact baseline research. While hypothesis-driven science proves a vital role, exploration and quantification of biodiversity and natural history are needed to both build theory-driven research and provide context for measuring impact and recovery. In this case, the lack of quantitative pre-spill data on Gulf megafauna in general specifically presents a major data gap and obstacle for determining what restored conditions may and should be. This lack of baseline data for deep-sea communities represents a major obstacle in the conservation and restoration of these systems both in the Gulf and globally [6,72].

Third, we urge for stronger and more explicit policy on monitoring efforts. As advocated recently by leading experts in deep-sea science [73], ‘A sustainable management strategy for the deep ocean should establish science-based conservation goals, develop a global framework for defining baseline conditions and establish monitoring requirements. Such a strategy must include objectives and definitions of key variables and indicators. It must consider the spatiotemporal frequency of biological data sampling necessary to document the ecological heterogeneity and status of the seabed and water column at depth. A global deep-ocean monitoring strategy would … protect and restore deep-sea ecosystems’. In general, conservation, monitoring and restoration of Earth's largest habitat, the deep oceans, should not be reactive and haphazard but proactive and mission-driven.

## Supplementary Material

Appendix

Reviewer comments

## Supplementary Material

Appendix

## Data accessibility

Data available at https://github.com/crmcclain/dwh.
