# Supplementary material for: Persistent and substantial impacts of the Deepwater Horizon oil spill on deep-sea megafauna
Source: R Soc Open Sci. 2019 Aug 28;6(8):191164. doi: 10.1098/rsos.191164 (PMC6731716; doi:10.1098/rsos.191164)
Supplement: Appendix [file rsos191164supp1.docx]

**Methods**

**Sample Areas**

**Supplemental Table 1:** Navigation information of transects conducted at Background, DWH 500_M North and DWH 2000M South sites in 2017.

| Transect | Date | Dive # | Site | Lease Block | Start Latitude | Start Longitude | Heading | Start Depth (meters) | End Depth (meters) |
| --- | --- | --- | --- | --- | --- | --- | --- | --- | --- |
| T1 | 5/26/2017 | GE1 | Background 1 | GC833 | 27.13516 | -89.92792 | 9 | 2179 | 2178 |
| T2 | 5/26/2017 | GE1 | Background 1 | GC833 | 27.13567 | -89.92766 | 9 | 2178 | 2176 |
| T3 | 5/26/2017 | GE1 | Background 1 | GC833 | 27.13762 | -89.92710 | 14 | 2176 | 2176 |
| T4 | 5/26/2017 | GE1 | Background 1 | GC833 | 27.13922 | -89.92642 | 20 | 2176 | 2174 |
| T5 | 5/27/2017 | GE2 | Background 1 | GC833 | 27.13649 | -89.92692 | 180 | 2168 | 2168 |
| T6 | 5/27/2017 | GE2 | Background 1 | GC833 | 27.13616 | -89.92673 | 185 | 2167 | 2170 |
| T7 | 5/28/2017 | GE3 | Background 1 | AT681 | 27.31281 | -88.92696 | 180 | 2036 | 2034 |
| T8 | 5/28/2017 | GE3 | Background 2 | AT681 | 27.31252 | -88.92771 | 90 | 2034 | 2034 |
| T9 | 5/28/2017 | GE3 | Background 2 | AT681 | 27.74563 | -88.92417 | 250 | 2033 | 2033 |
| T10 | 5/29/2017 | GE4 | Background 3 | MC867 | 28.10621 | -88.45050 | 180 | 1984 | 1985 |
| T11 | 5/28/2017 | GE4 | Background 3 | MC867 | 28.10574 | -88.45064 | 270 | 1984 | 1985 |
| T12 | 5/29/2017 | GE4 | Background 3 | MC867 | 28.10564 | -88.45158 | 40 | 1983 | 1984 |
| T13 | 5/29/2017 | GE4 | Background 3 | MC867 | 28.10616 | -88.45110 | 125 | 1985 | 1984 |
| T14 | 5/29/2017 | GE4 | Background 3 | MC867 | 28.10644 | -88.44876 | 150 | 1986 | 1986 |
| T15 | 5/29/2017 | GE4 | Background 3 | MC867 | 28.10655 | -88.44795 | 280 | 1985 | 1986 |
| T16 | 5/31/2017 | GE6 | Background 4 | DC134 | 28.81059 | -88.81040 | 160 | 1960 | 1964 |
| T17 | 5/31/2017 | GE6 | Background 4 | DC134 | 28.81147 | -88.81479 | 270 | 1962 | 1963 |
| T18 | 6/1/2017 | GE7 | 500_M North | MC252 | 28.74353 | -88.36863 | 90 | 1501 | 1506 |
| T19 | 6/1/2017 | GE7 | 500_M North | MC252 | 28.74311 | -88.36640 | 90 | 1506 | 1511 |
| T20 | 6/1/2017 | GE7 | 500_M North | MC252 | 28.74411 | -88.36361 | 247 | 1508 | 1506 |
| T21 | 6/1/2017 | GE7 | 500_M North | MC252 | 28.74328 | -88.36630 | 292 | 1506 | 1499 |
| T22 | 6/1/2017 | GE7 | 500_M North | MC252 | 28.74483 | -88.36789 | 135 | 1498 | 1506 |
| T23 | 6/1/2017 | GE7 | 500_M North | MC252 | 28.74313 | -88.36617 | 45 | 1505 | 1505 |
| T24 | 6/1/2017 | GE7 | 500_M North | MC252 | 28.74542 | -88.36504 | 202.5 | 1503 | 1507 |
| T25 | 6/1/2017 | GE7 | 500_M North | MC252 | 28.74333 | -88.36588 | 337.5 | 1507 | 1501 |
| T26 | 6/1/2017 | GE7 | 500_M North | MC252 | 28.74572 | -88.36616 | 180 | 1500 | 1506 |
| T27 | 6/2/2017 | GE8 | 500_M North | MC252 | 28.72258 | -88.36529 | 180 | 1594 | 1597 |
| T28 | 6/2/2017 | GE8 | 500_M North | MC252 | 28.71965 | -88.36529 | 180 | 1597 | 1601 |
| T29 | 6/2/2017 | GE8 | 2000_M South | MC252 | 28.71880 | -88.36727 | 45 | 1597 | 1595 |
| T30 | 6/2/2017 | GE8 | 2000_M South | MC252 | 28.72059 | -88.36543 | 45 | 1595 | 1594 |
| T31 | 6/2/2017 | GE8 | 2000_M South | MC252 | 28.72048 | -88.36303 | 270 | 1597 | 1596 |
| T32 | 6/2/2017 | GE8 | 2000_M South | MC252 | 28.72032 | -88.36551 | 270 | 1596 | 1596 |
| VB1 | 8/19/2010 | VB3 | 2000_M South | MC252 | 28.72031 | -88.36572 | 0 | 1585 | 1591 |
| VB2 | 8/19/2010 | VB3 | 2000_M South | MC252 | 28.72039 | -88.36568 | 45 | 1585 | 1591 |
| VB3 | 8/19/2010 | VB3 | 2000_M South | MC252 | 28.72034 | -88.36572 | 60 | 1585 | 1591 |
| VB4 | 8/19/2010 | VB3 | 2000_M South | MC252 | 28.72034 | -88.36566 | 75 | 1585 | 1591 |
| VB5 | 8/19/2010 | VB3 | 2000_M South | MC252 | 28.72041 | -88.36484 | 90 | 1585 | 1591 |
| VB6 | 8/19/2010 | VB3 | 2000_M South | MC252 | 28.72032 | -88.36564 | 105 | 1585 | 1591 |
| VB7 | 8/19/2010 | VB3 | 2000_M South | MC252 | 28.72048 | -88.36587 | 120 | 1585 | 1591 |
| VB8 | 8/19/2010 | VB3 | 2000_M South | MC252 | 28.72040 | -88.36581 | 135 | 1585 | 1591 |
| VB9 | 8/19/2010 | VB3 | 2000_M South | MC252 | 28.72036 | -88.36575 | 165 | 1585 | 1591 |
| VB10 | 8/19/2010 | VB3 | 2000_M South | MC252 | 28.72033 | -88.36571 | 180 | 1585 | 1591 |
| VB11 | 8/19/2010 | VB3 | 2000_M South | MC252 | 28.72048 | -88.36624 | 225 | 1585 | 1591 |
| VB12 | 8/19/2010 | VB3 | 2000_M South | MC252 | 28.72033 | -88.36555 | 240 | 1585 | 1591 |
| VB13 | 8/19/2010 | VB3 | 2000_M South | MC252 | 28.72031 | -88.36582 | 255 | 1585 | 1591 |
| VB14 | 8/19/2010 | VB3 | 2000_M South | MC252 | 28.72040 | -88.36603 | 270 | 1585 | 1591 |
| VB15 | 8/19/2010 | VB3 | 2000_M South | MC252 | 28.72031 | -88.36572 | 285 | 1585 | 1591 |
| VB16 | 8/19/2010 | VB3 | 2000_M South | MC252 | 28.72047 | -88.36588 | 300 | 1585 | 1591 |
| VB17 | 8/19/2010 | VB3 | 2000_M South | MC252 | 28.72042 | -88.36580 | 315 | 1585 | 1591 |
| VB18 | 8/19/2010 | VB3 | 2000_M South | MC252 | 28.72034 | -88.36579 | 330 | 1585 | 1591 |
| VB19 | 8/19/2010 | VB3 | 2000_M South | MC252 | 28.74322 | -88.36609 | 0 | 1585 | 1591 |
| VB20 | 8/21/2010 | VB5 | 500_M North | MC252 | 28.74321 | -88.36606 | 22.5 | 1493 | 1509 |
| VB21 | 8/21/2010 | VB5 | 500_M North | MC252 | 28.74325 | -88.36606 | 45 | 1493 | 1509 |
| VB22 | 8/21/2010 | VB5 | 500_M North | MC252 | 28.74324 | -88.36606 | 67.5 | 1493 | 1509 |
| VB23 | 8/21/2010 | VB5 | 500_M North | MC252 | 28.74324 | -88.36613 | 90 | 1493 | 1509 |
| VB24 | 8/21/2010 | VB5 | 500_M North | MC252 | 28.74320 | -88.36611 | 270 | 1493 | 1509 |
| VB25 | 8/21/2010 | VB5 | 500_M North | MC252 | 28.74323 | -88.36606 | 292.5 | 1493 | 1509 |
| VB26 | 8/21/2010 | VB5 | 500_M North | MC252 | 28.74342 | -88.36630 | 315 | 1493 | 1509 |

**Analytical Methods**

Alpha megafauna diversity was calculated for video transect using the Shannon–Wiener index as defined as *H = -sum p_i_ ln(p_i_)*_,_ where *p_i_* is the proportional abundance of species *i*. *H* was calculated using the vegan package (1) in R. Rarefaction curves were calculated for combined transects for each site*time combination using the iNEXT package (2, 3) with interpolation and extrapolation of Hill number with diversity order equal to one. Abundance was the total number individuals per transect. Linear Mixed-Effects Models were constructed with the lmer function in the package lme4 (4). ANOVAs were analyzed with the Anova function in the package car (5). In each ANOVA, transect group—DWH 2010, Background 2017, and DWH 2017— was the independent variable with *H* or abundance as the dependent. Sites within groups were treated as random effects in the models. *Post-hoc* tests were conducted using Tukey Honest Significant Differences test with the ghlt function in the package multicomp (6).

Total beta diversity was calculated on the Hellinger-transformed abundance as the total variance in a community data matrix (7) using the adespatial package (8) in R. The Hellinger transformation was implemented to preserve Euclidean distances between transects as advocated for when in use with redundancy or principal components analysis (9). Hellinger distance also offered a better compromise between linearity and resolution than some other distance metrics (9). Total beta diversity was calculated for groups of transects including all 2010 DWH, DWH 2010 500-N, DWH 2010 2000-S, all 2017 DWH, DWH 2017 500-N, DWH 2017 2000-S, Background Sites 1, 2, 3, and 4. Total beta diversity was calculated as the total variance in a community data matrix.

A principle component analysis was conducted on the Hellinger pre-transformed data, using the function deconstand and rda in the vegan package (1). Compositional differences between groups were tested using the PERMDSP2 procedure for the analysis of multivariate homogeneity of group dispersions (variances) using the betadispr function in the vegan package (1) with a subsequent permutation test. In this method, the average multivariate distance of individual transects is calculated from the group centroid in the PCA space. To test if the dispersions of the three groups—DWH 2010, Background 2017, and DWH 2017—were different, these distances were analyzed with ANOVA.

*C. quinquidens* raw data were turned into a Gower’s distance matrix and analyzed in multivariate space using a principle coordinates analysis with vegdist and cmdscale in the vegan package (1). Differences between groups—pre-oil spill, post-oil spill DWH site, and post-oil spill background-were analyzed using the PERMDSP2 procedure for the analysis of multivariate homogeneity of group dispersions (variances) using the betadispr function in the vegan package (1) with a subsequent permutation test. In this method, the average multivariate distance of the three groups was calculated from the group centroid in the PCA space. To test if the dispersions of the three groups were different, these distance were analyzed with ANOVA.

**Supplemental Table 2:** Health Codings for *Chaceon quinquidens* at the Deep-Water Horizon site

| Trait | Description |
| --- | --- |
| Barnacle Number | Total number of ectoparasitic barnacles visible on crab. 999 is NA. |
| Defensive | Whether the crab shows a defensive behavior defined as the crab placing claws up and towards the ROV in a defensive position. 0 is no |
| Retreat | Whether the crab shows a retreating behavior defined as the crab moving away from ROV. |
| Color | Color of the crab. RED means that the crab is a majority red. BLACK means that the crab is majority black. MIX means that the crab is approximately 50% red and 50% black. 999 is NA. |
| Missing Number of Legs | The total number of legs the crab is missing. 999 is NA. |
| Deformities | Denotes if the crab has any deformities on claws, legs, or carapace. 0 is no and 1 is yes. |
| Claws Present | Total claws present on the crab. 0,1, or 2. 999 is NA. |

**Supplemental Table 3:** ANOVA for Group~Shannon H’ for megafauna observed along ROV transects. Groups are DWH 2010, DWH 2017, and Background 2017.

Linear mixed model fit by REML ['lmerMod']

Formula: H ~ Group + +(1 | Site)

Data: DWH

REML criterion at convergence: 101.1

**Scaled residuals:**

Min 1Q Median 3Q Max

-1.5410 -1.0307 -0.1475 0.6591 3.1879

**Random effects:**

Groups Name Variance Std.Dev.

Site (Intercept) 0.002673 0.0517

Residual 0.285437 0.5343

Number of obs: 61, groups: Site, 6

**Fixed effects:**

Estimate Std. Error t value

(Intercept) 1.2131 0.1326 9.151

GroupDWH2010 -0.6487 0.1721 -3.770

GroupDWH2017 -0.2032 0.1890 -1.075

**Response: H**

Chisq Df Pr(>Chisq)

Group 16.092 2 0.0003204

**Supplemental Table 4:** Posthoc multiple comparisons of means for Group~Shannon H’ for megafauna observed along ROV transects.

**Estimate Std. Error z value Pr(>|z|)**

DWH2010 - 2017Background == 0 -0.6487 0.1721 -3.770 **<0.001**

DWH2017 - 2017Background == 0 -0.2032 0.1890 -1.075 0.5285

DWH2017 - DWH2010 == 0 0.4456 0.1659 2.685 **0.0197**

**Supplemental Table 5:** Permutation test for homogeneity of multivariate dispersions of megafauna transects using Hellinger transformed data

| Df | Df | Sum of Squares | Mean Sum of Squares | F values | P-value |
| --- | --- | --- | --- | --- | --- |
| Group | 2 | 2.008 | 1.00399 | 39.104 | 0.001 |
| Residuals | 50 | 1.2837 | 0.02567 |  |  |

**Supplemental Table 6:** Pairwise comparisons of megafauna transects using Hellinger transformed data. Observed p-value below diagonal, permuted p-value above diagonal.

|  | 2017Background | DWH2010 | DWH2017 |
| --- | --- | --- | --- |
| 2017Background |  | <0.0001 | 0.001 |
| DWH2010 | <0.0001 |  | 0.001 |
| DWH2017 | <0.0001 | <0.0001 |  |

**Supplemental Table 7:** ANOVA for Group~Abundance for megafauna observed along ROV transects. Groups are DWH 2010, DWH 2017, and Background 2017.

Linear mixed model fit by REML ['lmerMod']

Formula: Total.Abundance ~ Group + +(1 | Site)

Data: DWH

REML criterion at convergence: 458.5

**Scaled residuals:**

Min 1Q Median 3Q Max

-2.5881 -0.2264 -0.0540 0.2056 4.0495

**Random effects:**

Groups Name Variance Std.Dev.

Site (Intercept) 2.812 1.677

Residual 134.574 11.601

Number of obs: 61, groups: Site, 6

**Fixed effects:**

Estimate Std. Error t value

(Intercept) 6.838 2.956 2.314

GroupDWH2010 -3.374 3.903 -0.864

GroupDWH2017 44.348 4.250 10.435

**Response: Total.Abundance**

Chisq Df Pr(>Chisq)

Group 192.23 2 < 2.2e-16

**Supplemental Table 8:** Posthoc multiple comparisons of means for Group~Abundance for megafauna observed along ROV transects.

Estimate Std. Error z value Pr(>|z|)

DWH2010 - 2017Background == 0 -3.374 3.903 -0.864 0.662

DWH2017 - 2017Background == 0 44.348 4.250 10.435 <1e-05

DWH2017 - DWH2010 == 0 47.722 3.613 13.209 <1e-05

**Supplemental Table 9:** ANOVA for Group~Abundance (without Arthropods) for megafauna observed along ROV transects. Groups are DWH 2010, DWH 2017, and Background 2017.

Linear mixed model fit by REML ['lmerMod']

Formula: Total.Abundace2 ~ Group + +(1 | Site)

Data: DWH

REML criterion at convergence: 292.5

**Scaled residuals:**

Min 1Q Median 3Q Max

-1.269 - 0.869 -0.132 0.730 2.194

**Random effects:**

Groups Name Variance Std.Dev.

Site (Intercept) 0.8641 0.9296

Residual 7.4697 2.7331

Number of obs: 61, groups: Site, 6

**Fixed effects:**

Estimate Std. Error t value

(Intercept) 5.0980 0.8265 6.168

GroupDWH2010 -2.2116 1.1858 -1.865

GroupDWH2017 -0.8557 1.2470 -0.686

**Response: Total.Abundance2**

Chisq Df Pr(>Chisq)

Group 4.6643 2 0.09708

**Supplemental Table 10:** Posthoc multiple comparisons of means for Group~Abundance (without arthropods) for megafauna observed along ROV transects.

**Estimate Std. Error z value Pr(>|z|)**

DWH2010 - 2017Background == 0 -2.2116 1.1858 -1.865 0.145

DWH2017 - 2017Background == 0 - 0.8557 1.2470 -0.686 0.768

DWH2017 - DWH2010 == 0 1.3558 0.8578 1.581 0.249

**Supplemental Table 11:** Principal coordinate axis loadings for health codings for *Chaceon quinquiden*

| Axis | Percent Variation Described | Cumulative Variance Described |
| --- | --- | --- |
| 1 | 11.44% | 11.44% |
| 2 | 7.21% | 18.65% |
| 3 | 4.12% | 22.78% |
| 4 | 4.01% | 26.79% |
| 5 | 3.40% | 30.19% |
| 6 | 2.62% | 32.81% |
| 7 | 2.20% | 35.01% |
| 8 | 1.88% | 36.90% |
| 9 | 1.43% | 38.33% |
| 10 | 0.62% | 38.95% |
| 11 | 0.52% | 39.47% |
| 12 | 0.38% | 39.85% |
| 13 | 0.33% | 40.18% |
| 14 | 0.32% | 40.50% |
| 15 | 0.32% | 40.82% |
| 16 | 0.32% | 41.14% |
| 17 | 0.32% | 41.46% |
| 18 | 0.32% | 41.78% |
| 19 | 0.32% | 42.10% |
| 20 | 0.32% | 42.42% |

**Supplemental Table 12:** Permutation test for homogeneity of multivariate dispersions of *Chaceon quinquidens* health using Gower Distance.

| Df | Df | Sum of Squares | Mean Sum of Squares | F values | P-value |
| --- | --- | --- | --- | --- | --- |
| Group | 2 | 0.07834 | 0.03917 | 6.3976 | 0.0040 |
| Residuals | 204 | 1.24902 | 0.006123 |  |  |

**Supplemental Table 13:** Pairwise comparisons of *Chaceon quinquidens* health using Gower Distance. Observed p-value below diagonal, permuted p-value above diagonal.

|  | Pre Oil Spill | Post Oil Spill Background | Post Oil Spill DWH |
| --- | --- | --- | --- |
| Pre Oil Spill |  | 0.0040 | 0.0020 |
| Post Oil Spill Background | 0.0012 |  | 0.2318 |
| Post Oil Spill DWH | 0.0012 | 0.2024 |  |

**Potential Depth Bias of Results**

One potential explanation for the differences observed here in 2017 between the background and the DWH sites is depth differences between the sites. The transects at Deep-Water Horizon range in depth from 1499-1591m and the background transects are 1984-2178 for a minimum of 393m and a maximum of 679m between the two groups. This sample design was an unfortunate outcome reflects that the research was unfunded and the dives at Deep-Water Horizon were opportunistic. The background ROV transects were collected as part of another supported research project. However, the depth difference is unlikely to explain the results.

First, the deeper background sites should exhibit lower, not the observed higher, alpha-diversity compared to the shallower DWH horizon sites (10). Indeed, the high particulate organic carbon flux of this Gulf of Mexico region (10, 11) should also drive diversity to be higher than the other deeper background sites (12-14).

Second, the abundances would be expected to be higher at the shallower sites (15, 16). And although abundance are higher at the shallower DWH site, increases in abundance would be expected to occur across megafaunal taxa. The result here show increases are limited to arthropods. The differences in abundances between background and impacted sites once are nonexistent when arthropods are removed. This finding suggests that abundance was impacted by a process other than a simple bathymetric and energy availability relationship.

Third, the substantial differences in taxonomic composition between the background and impacted sites suggest as well a mechanism beyond bathymetric differences. The 2017 background and Deep-Water Horizon communities are less than 20% similar in composition to each other. The ~500m depth difference between DWH and background sites is unlikely to account for a >80% compositional change. This turnover percentage is more typical of a greater than 1000m depth difference at much shallower depths (17). Upper to lower middle slope communities often show >40% similarity (18). Indeed, research from the Gulf of Mexico indicates the compositional differences in communities at this depth and over this depth range would be minimal. Powell et al (19) demonstrate that the deep-sea demersal fish of the Northern Gulf of Mexico form a single compositional group from depths 1533-3075m based on Bray-Curtis similarities and cluster analysis. Pequegnat et al.’s (20) classic paper shows that the megafauna of the Gulf of Mexico fall naturally into several depth zones including the “Upper Abyssal Zone” from 1,000-2,275. These combined lines of evidence indicated the bathymetric differences between the Deep-Water Horizon and background transects are insufficient to account for the differences observed here.

**Literature Cited**

1. Oksanen J*, et al.* (2013) vegan: Community Ecology Package), R package version 2.0-9.

2. Chao A*, et al.* (2014) Rarefaction and extrapolation with Hill numbers: a framework for sampling and estimation in species diversity studies. *Ecological Monographs* 84:45-67.

3. Hsieh TC, Ma KH, & Chao A (2018) iNEXT: iNterpolation and EXTrapolation for species diversity).

4. Bates D, Mächler M, Bolker B, & Walker S (2015) Fitting Linear Mixed-Effects Models Using {lme4}. *Journal of Statistical Software* 67:1-48.

5. Fox J & Weisberg S (2011) An {R} Companion to Applied Regression. (Sage, Thosand Oaks, CA).

6. Hothorn T, Bretz F, & Westfall P (2008) Simultaneous inference in general parametric models. *Biometrical Jounral* 50:346-363.

7. Pierre L & Miquel C (2013) Beta diversity as the variance of community data: dissimilarity coefficients and partitioning. *Ecology Letters* 16(8):951-963.

8. Dray S*, et al.* (2018) despatial: Multivariate Multiscale Spatial Analysis), R package version 0.1-1.

9. Legendre P & Gallagher ED (2001) Ecologically meaningful transformation for ordination of species data. *Oecologia* 129:271-280.

10. Wei C-L*, et al.* (2010) Bathymetric zonation of deep-sea macrofauna in relation to export of surface phytoplankton production. *Marine Ecology Progress Series* 399:1-14.

11. Wei C-L*, et al.* (2012) Standing stocks and body size of deep-sea macrofauna: Predicting thebaseline of 2010Deepwater Horizonoil spill in the northern Gulf of Mexico. *Deep Sea Research Part I: Oceanographic Research Papers* 69:82-99.

12. Tittensor DP, Rex MA, Stuart CT, McClain CR, & Smith CR (2011) Species-energy relationships in deep-sea mollusks. *Biology Letters* 7:718-722.

13. Woolley SNC*, et al.* (2016) Deep-sea diversity patterns are shsaped by energy availability. *Nature* 533:393-396.

14. McClain CR, Allen AP, Tittensor DP, & Rex MA (2012) The energetics of life on the deep seafloor. *Proceedings of the National Academy of Science, U.S.A.* 109:5366–15371.

15. Wei C-L, Rowe G, Escobar-Briones E, & Boetius A (2010) Global Patterns and Predictions of Seafloor Biomass Using Random Forests. *PLoS ONE*.

16. Rex MA*, et al.* (2006) Global bathymetric patterns of standing stock and body size in the deep-sea benthos. *Marine Ecology Progress Series* 317:1-8.

17. Rex MA (1981) Community structure in the deep-sea benthos. *Annual Review of Ecology and Systematics* 12:331-353.

18. Hecker B (1990) Variation in megafaunal assemblages on the continental margin south of New England. *Deep Sea Research Part A. Oceanographic Research Papers* 37(1):37-57.

19. Powell SM, Haedrich RL, & McEachran JD (2003) The deep-sea demersal fish fauna of the northern Gulf of Mexico. *Journal of Northwest Atlantic Fishery Science* 31:19-33.

20. Pequegnat WE, Gallaway BJ, & Pequegnat LH (1990) Aspects of the ecology of the deep-water fauna of the Gulf of Mexico. *American Zoologist* 30(1):45-64.
