# Supplementary material for: Persistent and substantial impacts of the Deepwater Horizon oil spill on deep-sea megafauna
Source: R Soc Open Sci. 2019 Aug 28;6(8):191164. doi: 10.1098/rsos.191164 (PMC6731716; doi:10.1098/rsos.191164)
Supplement: Reviewer comments [file rsos191164_review_history.pdf]

THE ROYAL SOCIETY
PUBLISHING

ROYAL SOCIETY
OPEN SCIENCE

Persistent and substantial impacts of the Deepwater Horizon oil spill on deep-sea megafauna

Craig R. McClain, Clifton Nunnally and Mark C. Benfield

Article citation details
R. Soc. open sci. **6**: 191164.
http://dx.doi.org/10.1098/rsos.191164

Review timeline
Original submission: 5 July 2019
Final acceptance: 17 July 2019

Note: Reports are unedited and appear as submitted by the referee. The review history appears in chronological order.

Note: This manuscript was transferred from another Royal Society journal with peer review.

Review History

RSOS-191164.R0 (Original submission)

Decision letter (RSOS-191164.R0)

17-Jul-2019

Dear Dr McClain:

It is a pleasure to accept your manuscript entitled "Persistent and substantial impacts of the Deepwater Horizon oil spill on deep-sea megafauna." in its current form for publication in Royal Society Open Science.

Thank you for your fine contribution. On behalf of the Editors of Royal Society Open Science, we look forward to your continued contributions to the Journal.

Reports © 2019 The Reviewers; Decision Letters © 2019 The Reviewers and Editors; Responses © 2019 The Reviewers, Editors and Authors. Published by the Royal Society under the terms of the Creative Commons Attribution License http://creativecommons.org/licenses/by/4.0/, which permits unrestricted use, provided the original author and source are credited

Best regards,

Lianne Parkhouse
Editorial Coordinator
Royal Society Open Science
openscience@royalsociety.org

on behalf of Dr Jenny Collier (Associate Editor) and Dr Kevin Padian (Subject Editor).

Associate Editor Dr Jenny Collier Comments to Author:

Having read all the documentation about this manuscript, and importantly your response to the reviewers I am happy to accept it for publication in RSOS without further review. I think it is an important, timely and provocative work that would make a good contribution to our journal.

Follow Royal Society Publishing on Twitter: @RSocPublishing
Follow Royal Society Publishing on Facebook:
https://www.facebook.com/RoyalSocietyPublishing.FanPage/
Read Royal Society Publishing's blog: https://blogs.royalsociety.org/publishing/
